# Prevalence and Factors Associated with Postoperative Nausea and Vomiting in an Ethiopian Comprehensive Specialized Hospital

**DOI:** 10.1155/2024/6699732

**Published:** 2024-02-16

**Authors:** Diriba Teshome, Metages Hunie, Simegnew Kibret, Marifa Mestofa, Efrem Fenta

**Affiliations:** Department of Anesthesia, School of Medicine, College of Health Sciences, Debre Tabor University, Debre Tabor, Ethiopia

## Abstract

**Background:**

Postoperative nausea and vomiting (PONV) is a common and uncomfortable anesthetic and surgical consequences. It may cause severe distress to the patient and may cause the recovery process to be delayed. Identifying the reasons may aid in reducing the magnitude and problems. The purpose of this study was to determine the prevalence and risk factors for PONV after general anesthesia in an Ethiopian hospital.

**Methods:**

From March 1 to May 30, 2019, a cross-sectional study was designed. A patient interview was used to obtain data on the occurrence of PONV, and a chart review was used to collect data on other demographic and clinical variables. To identify associated factors, variables with a *P*-value of 0.2 in binary logistic regression were transformed into a multivariable logistic regression. The strength of the association and level of significance waswere demonstrated using crude and adjusted odds ratios with 95% confidence intervals and *P*-values of 0.05.

**Results:**

The study included 162 participants, with a remarkable 100% response rate. Within 24 hr after surgery, 51.2% of patients had nausea and vomiting. When compared to their counterparts, female patients, patients who received perioperative opioid medication, patients with a history of PONV, and patients with a history of motion sickness reported a statistically significant difference (higher incidence) in PONV.

**Conclusion:**

This study only comprised ASA physical classes 1 and 2 patients who did not receive preventive antiemetics. In the research area, the total prevalence of vomiting and nausea was 51.2%. Female sex, perioperative opioid usage, a history of nausea and vomiting, and a history of motion sickness were discovered to be statistically significantly associated with a higher incidence of PONV.

## 1. Introduction

Postoperative nausea and vomiting (PONV) is defined as nausea and vomiting occurring within 24 hr after surgery. It is the most common and unpleasant complication of general anesthesia and surgery [1]. Nausea is defined as a sensation associated with awareness of the urge to vomit, accompanied by gastrointestinal tract relaxation, duodenal peristalsis, and vegetative symptoms [2].

The central nervous system areas which control balance, vasomotor activity, salivation, respiration, and eye motion are located close to the vomiting center and they are interconnected. The proximity of these areas is responsible for physiological vegetative reactions observed in PONV, such as salivation, sweating, frequent gulping, pallor, tachypnea, tachycardia, heart rhythm disturbance, pupil dilation, and motion sickness [3, 4].

Vomiting, the forceful expulsion of stomach contents through the mouth, is a neurologically conducted, coordinated reflex in which visceral reflexes in the medulla oblongata are integrated, including their coordination and time synchronization with somatic components [5].

Nausea and vomiting in the postoperative period can cause reduced patient comfort, delayed discharge, and increased cost of care. It could cause dehydration, electrolyte imbalance, venous hypertension, wound dehiscence, bleeding, rupture of the esophagus, airway obstruction, and aspiration pneumonia [6–8].

PONV is a long-standing, most unpleasant, and multifactorial problem for anesthesia practitioners with significant and most distressing morbidities, associated with surgery and anesthesia [4].

The overall incidence of PONV is estimated to be 28%–80%, with severe, intractable PONV estimated to occur in approximately 18% of all patients undergoing surgery [9–14]. Around 2.4 million patients suffer from PONV every year in Germany where better medical care is expected to be provided as compared to a low-resource settings like Ethiopia [6, 15].

The overall prevalence of PONV in surgical patients is 25%–30% in the American Society of Anesthesiologists (ASA) 1 and 2 patients, but for ASA 3 and above patients (among high-risk patients), it could be as high as 70%–80% [16]. The incidence of PONV remains high despite the frequent use of prophylactic antiemetics [17].

Multiple factors are associated with increased risk of PONV: age, gender, preexisting disease, premedication, history of PONV, operative procedure, anesthetics agents, analgesic drugs, anesthetics duration, and postoperative morbidities [18].

This study helps to identify the prevalence and associated factors of PONV in generating management strategies to reduce its occurrence and establish baseline information for future researchers and health program planners. This study aimed to assess the prevalence and associated factors of PONV under general anesthesia at Debre Tabor Comprehensive Specialized Hospital (DTCSH).

## 2. Methods

Study design, setting, and period: Hospital-based cross-sectional study was conducted at DTCSH which is located in the Debub Gondar Zone of the Amhara Regional State of Ethiopia, about 100 km northeast of Bahir Dar city and 50 km east of Lake Tana, with a latitude and longitude of 11°51′N 38°1′E and an elevation of 2,706 m (8,878 ft) above sea level [19]. The Hospital provides more than 2,000 surgical cases annually. The study was conducted from March 1 to May 30, 2019.

### 2.1. Source Population

All adult elective surgical patients scheduled for surgery under general anesthesia in DTCSH.

### 2.2. Study Population

Elective surgical patients scheduled for surgery under general anesthesia who met inclusion criteria during the study period in DTCSH.

### 2.3. Inclusion Criteria

Patient age 18 and above, ASA 1 and 2, and underwent elective surgery under general anesthesia.

### 2.4. Exclusion Criteria

Patients who received antiemetic medication before surgery were excluded.

### 2.5. Dependent Variable

Postoperative nausea and vomiting (yes/no).

### 2.6. Independent Variable

Independent variable are as follows: age, sex, body mass index (BMI), American Society of Anesthesiologist (ASA) physical status classification system, history of PONV, history of motion sickness, history of postoperative opioids usage, history of smoking, duration of surgery, duration of anesthesia, type of surgery, types of anesthetics agents, total blood loss, total intravenous fluid intake.

### 2.7. Operational Definition

PONV: The occurrence of nausea and vomiting in the first 24 hr after surgery.

### 2.8. Sampling Size and Sampling Technique

The sample size was calculated using the single population formula, from a previous study done at the University of Gondar, Ethiopia, prevalence of PONV of 36.2% [6]:

(1) $$\begin{array}{c} n=\frac{Z2\left(P\right)\left(1-P\right)}{d2}, \end{array}$$

where *n* is the sample size, *Z* is the confidence interval (1.96), *P* is the estimated prevalence (0.362), and *d* is the margin of sampling error to be tolerated (0.05).

To get the sampling size with a confidence interval of 95% and a margin of error 5%, the following equation is used:

(2) $$\begin{array}{c} n=\frac{{\left(1.96\right)}^{2}0.362\left(1-0.362\right)}{0.05^{2}}=355. \end{array}$$

By applying a finite population correction formula, the final sample size will be as follows:

(3) $$\begin{array}{c} \text{Nf}=\frac{n}{\left(1+n/N\right)}, \end{array}$$

where Nf is the final sample size, *n* is the sample size, and *N* is the total number of surgical procedures under GA for 3 months which was 275. Therefore, the final sample size (Nf) was 162 after adding a 5% nonresponse rate.

Systematic random sampling technique was used to select study participants from the operation schedule by using a skip interval of *K* = 275/162 which is approximately 2; where *n* is the total sampling size, *K* is the skip interval, and *N* is the total study population. The first study participant was selected by the lottery method and then every other patient was included.

### 2.9. Data Collection Procedure

Data were collected by anesthetists with structured questionnaires through chart review and patient interviews within 24 hr of the postoperative period. For the occurrence of PONV, we used the patient interview to avoid missing some cases as documentation could not be complete, especially in resource-limited settings like ours. Other demographic clinical variables are recorded from the chart.

### 2.10. Data Quality Control

Data were collected after training was given to data collectors. The collected data were properly filled in the prepared format. Then, the questionnaires were checked for accuracy, clarity, and consistency.

### 2.11. Data Management and Analysis

The data were coded and entered into the SPSS version 23 statistical package. Binary logistic regression analysis was used to identify potential associated factors between dependent and independent variables. Multivariate logistic regression analysis was used to determine the association of the combination of risk factors with PONV. All variables with a *P* ≤ 0.2 in univariate analysis were entered jointly into a multivariable logistic regression to check their association with the outcome variable. The odds ratio, 95% confidence interval, and *P*-value were computed to identify associated factors and to determine the strength of the association. A *P*-value of < 0.05 was considered statistically significant. Hosmer–Lemeshow test of goodness of fit was performed to check the appropriateness of the analysis model.

## 3. Results

### 3.1. Demographic Characteristics and Preoperative Conditions of Study Subjects

A total of 162 patients were included with a response rate of 100%. Out of 162 respondents of the study, 85 (52.5%) were males and 77 (47.5%) were female. The majority of the respondents 150 (92.6%) were classified in ASA 1. The majority of study participants have a history of motion sickness 90 (55.6%) (Table 1).

### 3.2. Intraoperative and Postoperative Surgical and Anesthetic Characteristics

Most of the respondents, 144 (88.9%) took general anesthesia with an endotracheal tube. Halothane was the only inhalational agent used for all study participants. The majority of patients 144 (88.9%) had the duration of surgery >120 min (Table 2).

Postoperative characteristics of the patients: 39% of patients developed nausea while 34% of the patients developed vomiting in PACU (Table 3).

The overall incidence of PONV: According to our results, 83 (51.2%) of the patients who underwent general anesthesia did experience nausea and vomiting within 24 hr after surgery.

The results of binary logistic regression analysis demonstrated the following characteristics were associated with the occurrence of PONV: sex, the administration of postoperative opioids, previous history of nausea and vomiting, and history of motion sickness.

In multiple logistic regression analyses, it was found that female patients who undergo GA were 5.6 times (AOR = 5.616; 95% CI: 2.580, 12.229) more likely to have PONV compared to male patients.

Patients who were given opioids postoperatively were 2.95 times (AOR = 2.951; 95% CI: 1.209, 7.202) more likely to have PONV compared to those who did not receive opioids.

In multilogistic regression patients, those who had a history of previous nausea and vomiting were 3.8 times (AOR = 3.822; 95% CI: 1.115, 13.107) more likely to have PONV compared to patients with no history of previous nausea and vomiting. Patients who had a history of motion sickness were 3.39 times (AOR = 3.386; 95% CI: 1.592, 7.199) more likely to have PONV compared to patients without a history of nausea and vomiting (Table 4).

## 4. Discussion

This Hospital-based cross-sectional study has attempted to assess the incidence and associated factors of PONV in an elective surgical patient under general anesthesia in DTCSH, Ethiopia.

This study found that the incidence of PONV among patients who underwent general anesthesia was 83 out of 162 (51.2%), which is similar to the results of previous studies [7, 13–15]. In contrast to our results, some studies report a lower incidence of PONV [6, 16–20]. The prevalence reported in this study is higher than the other previously reported studies in Ethiopia. The prevalence of PONV at Debre Berhan Referral Hospital was 21.8% [10], at the University of Gondar comprehensive specialized hospital was 17.2% [9], Jimma Medical Center was 27.4% [12], pediatric ophthalmic surgery at the University of Gondar comprehensive specialized hospital was 19.9% [11], and at Wolaita Sodo Teaching Referral Hospital was 29.1% [13] within 24 hr after the operation. This difference may be due to the use of different induction agents and in the current study population received the type of surgeries that have a higher PONV prevalence: abdominal, gynecological, and thyroid surgeries.

In our study, we found that being female, a history of previous PONV, a history of motion sickness, and patients who received postoperative opioids were significantly associated with PONV. Consistent with our findings, studies show that being female, having a history of previous PONV, having a history of motion sickness, and being patients premedicated with opioids were high-risk factors for PONV [1, 4, 6, 20, 21].

Apfel et al. [15] defined the risk criteria with the largest impact on PONV, and multiple other risk factors have been identified. These can be broadly divided into three categories: patient's risks (female gender, nonsmoking status, previous history of PONV/motion sickness, and genetic predisposition), anesthetics technique (inhalation agents, nitrous oxide, large-dose neostigmine, and intraoperative and postoperative opioids use), and surgical procedure (longer duration of surgery and different types of surgeries). However, whether longer surgeries are directly causal is difficult to prove since higher doses of opioids and longer exposure to inhalational anesthetics are likely to occur, as both are known risk factors for PONV [7, 15].

## 5. Conclusion

This study included only ASA physical classes 1 and 2 patients and in which patients did not receive prophylactic antiemetic. The overall prevalence of vomiting and nausea in the study area was 51.2%. The contributing factors were found to be female sex, preoperative opioid use, previous nausea and vomiting history, and history of motion sickness.

## 6. Recommendations

We strive to create awareness of PONV for health professionals who are involved in postoperative patient management at DTCSH. We suggest using these results to develop a protocol for the assessment and treatment of PONV in DTCSH.

## 7. What Is Known About This Topic?

PONV is a common problem for patients undergoing general anesthesia. It is well studied in developed countries but it is not well established in under-resourced settings like DTCSH.

## 8. What This Study Adds

The development of PONV is not well-studied in this practice setting. Identifying the risk factors associated with PONV provides the framework to develop assessment tools and management options.

## Figures and Tables

**Table 1:** Demographic characteristics and preoperative conditions of study participants at DTCSH from March 1 to May 30, 2019 (*N* = 162).

Variable		Frequency	Percentage (%)
Age			
18–24		9	5.6
25–29		60	37.0
30–34		51	31.5
35–39		30	18.5
40–44		6	3.7
45–49		4	2.5
50–65		2	1.2
Sex			
Male		85	52.5
Female		77	47.5
BMI (kg/m^2^)			
Below 18		6	3.7
18–24.9		144	88.9
25–29.9		12	7.4
ASA			
ASA 1		150	92.6
ASA 2		12	7.4
History of nausea and vomiting			
Yes		30	18.5
No		132	81.5
History of motion sickness			
Yes		90	55.6
No		72	44.4
History of smoking			
Yes		18	11.1
No		144	88.9

**Table 2:** Intraoperative surgical and anesthesia characteristics of study participants at DTCSH from March 1 to May 30, 2019 (*N* = 162).

Variable	Frequency	Percentage (%)
Type of anesthesia		
GA with Laryngeal mask airway	18	11.1
GA with Endotracheal tube	144	88.9
Induction agents		
Propofol	108	66.7
Ketamine	36	22.2
Thiopentone	18	11.1
Type of surgery		
Abdominal	78	48.1
Gynecology	51	31.5
Breast surgery	15	9.3
Thyroid	18	11.1
Duration of surgery (min)		
60–90	9	5.6
90–120	9	5.6
>120	144	88.9
Duration of anesthesia (min)		
60–90	8	4.9
90–120	10	6.2
>120	144	88.9
Total blood loss (ml)		
<250	6	3.7
250−500	48	29.6
500−750	66	40.7
750−1,000	42	25.9
Total fluid given intraoperatively (ml)		
250−500	3	1.9
500−1,000	45	27.8
1,000−2,000	105	64.8
>2,000	9	5.6
Postoperative opioids usage		
Yes	119	73.5
No	43	26.5

**Table 3:** Postoperative characteristics of study participants at DTCSH from March 1 to May 30, 2019 (*N* = 162).

Variable	Frequency	Percentage (%)
The occurrence of nausea in PACU		
Yes	63	38.9
No	99	61.1
The occurrence of vomiting in PACU		
Yes	55	34
No	107	66
The occurrence of nausea in the patient ward		
Yes	44	27.2
No	118	72.8
The occurrence of vomiting in the patient ward		
Yes	37	22.8
No	125	77.2
Occurrence period of postoperative nausea (hr)		
Early 6	55	34
6–24	36	22.2
Occurrence period of postoperative vomiting (hr)		
Early 6	50	30.8
6–24	33	20.4

**Table 4:** Factors associated with the occurrence of nausea and vomiting of study participants at DTCSH from March 1 to May 30, 2019 (*N* = 162).

Variable	PONV		COR (95% CI)	AOR (95% CI)	*P*-value
	Yes	No			
Sex					
Male	31	54	1	1	0.001
Female	52	25	3.623 (1.891, 6.941)	5.616 (2.580, 12.229)	
Previous history of nausea and vomiting					
No	61	71	1	1	0.02
Yes	22	8	3.265 (1.126, 9.465)	3.822 (1.115, 13.107)	
Postoperative opioids are given					
No	16	27	1	1	0.017
Yes	67	52	2.115 (1.039, 4.303)	2.951 (1.209, 7.202)	
History of motion sickness					
No	27	45	1	1	0.002
Yes	56	34	3.611 (1.879, 6.940)	3.386 (1.592, 7.199)	

## Data Availability

Data will be shared upon reasonable request from the corresponding author.

## Abbreviations

AOR: Adjusted odds ratio
ASA: American Society of Anesthesiology
CI: Confidence interval
COR: Crudes odds ratio
DTCSH: Debre Tabor Comprehensive Specialized Hospital
GA: General anesthesia
LMA: Laryngeal mask airway
PONV: Postoperative nausea and vomiting.

## References

[1] Murphy M. J., Hooper V. D., Sullivan E., Clifford T., Apfel C. C. (2006). Identification of risk factors for postoperative nausea and vomiting in the perianesthesia adult patient. *Journal of Peri Anesthesia Nursing*.

[2] Habib A. S., Chen Y.-T., Taguchi A., Henry Hu X., Gan T. J. (2006). Postoperative nausea and vomiting following inpatient surgeries in a teaching hospital: a retrospective database analysis. *Current Medical Research and Opinion*.

[3] Kovac A. L. (2000). Prevention and treatment of postoperative nausea and vomiting. *Drugs*.

[4] Doubravska L., Dostalova K., Fritscherova S., Zapletalova J., Adamus M. (2010). Incidence of postoperative nausea and vomiting in patients at a university hospital. Where are we today?. *Biomedical Papers*.

[5] Brady M. C., Kinn S., Stuart P., Ness V., Cochrane Wounds Group (2003). Preoperative fasting for adults to prevent perioperative complications. *Cochrane Database of Systematic Reviews*.

[6] Endale G/Egziabher G/medhn, Hoyle J., Reddi D., Melkie T. B. (2014). Prevalence and factors associated with postoperative nausea and vomiting at the university of Gondar teaching hospital, northwest Ethiopia, 2012: a cross-sectional study. *Ethiopian Journal of Health and Biomedical Sciences*.

[7] Chalya P. L., Mhewa O. Z., Mabula J. B. (2015). Postoperative nausea and vomiting at a tertiary care hospital in north-western Tanzania. *Tanzania Journal of Health Research*.

[8] Apfel C. C., Kranke P., Katz M. H. (2002). Volatile anaesthetics may be the main cause of early but not delayed postoperative vomiting: a randomized controlled trial of factorial design. *British Journal of Anaesthesia*.

[9] Ahmed S. A., Lema G. F. (2020). Incidence and factors associated with postoperative nausea and vomiting among elective adult surgical patients at University of Gondar comprehensive specialized hospital, northwest Ethiopia, 2019: a cross-sectional study. *International Journal of Surgery Open*.

[10] Allene M. D., Demsie D. G. (2020). Incidence and factors associated with postoperative nausea and vomiting at Debre Berhan referral hospital, NorthShewa, Ethiopia: across-sectional study. *International Journal of Surgery Open*.

[11] Aytolign H. A., Nigatu Y. A., Denu Z. A., Arefayine N. R. I. (2020). Incidence of post-operative nausea and vomiting and its associated factors following pediatric ophthalmic surgery at University of Gondar Comprehensive Specialized Hospital, 2019. *International Journal of Surgery Open*.

[12] Obsa M. S., Edosa D. C., Desalegn Z. M., Fanta N. D., Tamiru S. M., Mokenin G. T. (2020). Incidence of post-operative nausea and vomiting and it’s predictors among adult elective surgical patients at Jimma Medical Center, south west Ethiopia. *Research Square*.

[13] Temesgen A. S., Wolde G. D. (2020). Incidence and risk factors associated with post-operative nausea and vomiting in elective adult surgical patients at Wolaita Sodo Teaching Referral Hospital: an institutional based cross-sectional study. *Research Square*.

[14] Villeret I., Laffon M., Duchalais A., Blond M. H., Lecuyer A. I., Mercier C. (2002). Incidence of postoperative nausea and vomitingin paediatric ambulatory surgery. *Pediatric Anesthesia*.

[15] Apfel C. C., Läärä E., Koivuranta M., Greim C.-A., Roewer N. (1999). A simplified risk score for predicting postoperative nausea and vomiting conclusions from cross-validations between two centers. *Anesthesiology*.

[16] ASHP (1999). ASHP therapeutic guidelines on the pharmacologic management of nausea and vomiting in adult and pediatric patients receiving chemotherapy or radiation therapy or undergoing surgery. *American Journal of Health-System Pharmacy*.

[17] Gan T. J., Diemunsch P., Habib A. S. (2014). Consensus guidelines for the management of postoperative nausea and vomiting. *Anesthesia & Analgesia*.

[18] Rüsch D., Eberhart L. H., Wallenborn J., Kranke P. (2010). Nausea and vomiting after surgery under general anesthesia: an evidence-based review concerning risk assessment, prevention, and treatment. *Deutsches Ärzteblatt International*.

[19] Graham J. (2001). Debre tabor: wikipedia. https://en.wikipedia.org/wiki/Debre_Tabor.

[20] Ssebuufu R., Kakande I., Okello M. (2009). Post-operative nausea and vomiting at Mulago hospital. *East and Central African Journal of Surgery*.

[21] Weilbach C., Rahe-Meyer N., Raymondos K., Weissig A., Scheinichen D., Piepenbrock S. (2006). Postoperative nausea and vomiting (PONV): usefulness of the Apfel-score for identification of high risk patients for PONV. *Acta Anaesthesiologica Belgica*.

